# Draft Genome Sequence of *Bacillus stratosphericus* LAMA 585, Isolated from the Atlantic Deep Sea

**DOI:** 10.1128/genomeA.00204-13

**Published:** 2013-05-02

**Authors:** André Oliveira de Souza Lima, Alencar Cabral, Fernando Dini Andreote, Angélica Cavalett, Marcos Luiz Pessatti, Francisco Dini-Andreote, Marcus Adonai Castro da Silva

**Affiliations:** Center for Technological Earth and Sea Sciences, University of “Vale do Itajaí,” Itajaí-SC, Brazila;; Department of Soil Science, “Luiz de Queiroz” College of Agriculture, University of São Paulo, Piracicaba-SP, Brazilb

## Abstract

*Bacillus stratosphericus* LAMA 585 was isolated from the Mid-Atlantic-Ridge seafloor (5,500-m depth). This bacterium presents the capacity for cellulase, xylanase, and lipase production when growing aerobically in marine-broth media. Genes involved in the tolerance of oligotrophic and extreme conditions and prospection of biotechnological products were annotated in the draft genome (3.7 Mb).

## GENOME ANNOUNCEMENT

*Bacillus stratosphericus* was originally isolated from air samples (above 21 km of altitude). It is a Gram-positive endospore-forming bacterium that grows in temperatures ranging from 8 to 37°C (1). Recently, *B. stratosphericus* was also isolated from soils (2) and estuarine sediments (3). Here we report the draft genome of *Bacillus stratosphericus* LAMA 585, isolated from sea-floor sediments collected at 5,500 m depth at the Romanche Trench (equatorial region of the Atlantic Ocean) (00°26′18′′N, 17°03′57′′W).

The genome-sequencing data were obtained by an Illumina HiSeq2000 system (101-nucleotide paired-end read sequencing from a 400- to 500-bp genomic library constructed). Sequence data quality trimming and *de novo* assembly were performed using the CLC Genomics Workbench (CLC GW, v 5.5.2). Trimmed data encompassed a total of 25,708,384 reads (Phred >Q30 = 96.54%), which allowed the chromosomal genome assembly to be sorted into 18 contigs, with a total length of 3,697,972 bp (625-fold coverage), and a 7,671-bp plasmid (pBSt1), with 41.16% CG overall content. A total of 4,263 open reading frames (ORFs) were predicted by CLC GW, and 78.3% (3,347 ORFs) were functionally assigned by automated annotation using Blast2Go (v 2.6.0) (4). The gene density was 0.90 genes per kb with an average length of 959 bp per gene. Gene ontology (GO) classified functions for 2,288 predicted genes, encompassing biological processes (63.3%), cellular components (35.9%), and molecular functions (30.6%). Attribution of enzyme commission (EC) was obtained for 776 proteins, most of them (91.6%) with a unique number and others (8.0%) bifunctional.

KEGG pathways predicted the synthesis of all 20 essential amino acids and d-amino acids for peptidoglycan. Ammonia and sulfate have also been found to be putative nutritional sources for this bacterium, which nevertheless does not assimilate nitrate. The production of NAD^+^ is derived from l-aspartate, also used as precursor for pantothenate and coenzyme-A production. Some other metabolic pathways indicate the production by this bacterium of several biotechnologically interesting compounds. For instance, genes for the biosynthesis of siderophores, betaine, and β-lactamases were annotated. The cytochrome P450 was also detected, thus suggesting the processing of other substrates, such as xenobiotics, vitamins, fatty acids, steroids, and prostaglandins (5). In addition, the presence of glutathione transferase indicates the capability of this organism to metabolize recalcitrant compounds, such as benzopyrene, naphthalene, and aflatoxin. Moreover, also annotated was a complete assemblage of genes related to folate metabolism, derived from phenylalanine. As expected, genes related to the synthesis of cellulases (β-glucosidase and endo-glucanase), xylanases (endo-xylanase), lipases (lipase and esterase), and xylanase were also found, corroborating data previously published by our team (6).

The plasmid pSt1 presented an additional six putative genes: two of them related to essential metabolism, two involved in DNA binding and replication, and two with as-yet-unknown function. This plasmid shares similarities with the cryptic one described in *B. pumilus* (7).

### Nucleotide sequence accession numbers.

This whole-genome shotgun project has been deposited at DDBJ/EMBL/GenBank under the accession number APAS00000000. The version described in this paper is the first version, APAS00000000.1.
